# A path to recovery for overlooked populations and their unique challenges: integrating rehabilitation in palliative care for patients with substance use disorders

**DOI:** 10.3389/fresc.2024.1373857

**Published:** 2024-05-02

**Authors:** Annas Aljassem, Michael Spickler, Nandita Kapur

**Affiliations:** ^1^ Department of Physical Medicine and Rehabilitation, Oakland University William Beaumont School of Medicine, Royal Oak, MI, United States; ^2^ Department of Physical Medicine and Rehabilitation, Corwell Health William Beaumont University Hospital, Royal Oak, MI, United States

**Keywords:** palliative, substance use disorder, addiction, physical medicine and rehabilitation, rehabilitation

## Abstract

Palliative care is a growing medical specialty focusing on providing compassionate and holistic management for those facing life-threatening diseases. These patients frequently present with physical, functional, emotional, and psychosocial problems that require comprehensive interdisciplinary management. However, there is a substantial opportunity to improve care for patients in palliative care who also have a substance use disorder (SUD). These opportunities include direct provision of SUD treatments by specialist palliative care providers and the integration of physical medicine and rehabilitation services. The purpose of this article is to examine the misunderstood and underutilized interaction between palliative care and SUDs, as well as describing the unique opportunities provided by physical medicine and rehabilitation providers to achieve a patient's palliative care goals and optimize overall quality of life. Substance Use Disorder is a chronic, often relapsing, illness that is relevant to palliative care practice due to the potential for significant morbidity and mortality through organ failure, chronic infections, and overdose syndromes. In traditional palliative care practice, it has been observed that past or current SUD diagnoses are often left untreated, resulting in increased distress, and exacerbating an already complex medical situation. Furthermore, many of these patients also experience physical, functional, or psychosocial changes that, when left untreated, will worsen distress and quality of life. To provide more comprehensive and successful palliative care for patients with SUD, the authors recommend an increased emphasis on specialist palliative care training in SUD management, proactive integration of rehabilitation services into the palliative care team, and consistent advocacy for these steps in various arenas. Combined, these actions can improve the care team's ability to provide a holistic, patient-centered approach that can have substantial positive outcomes for patients, health systems, and society.

## Introduction

Substance use disorder (SUD) is a chronic, often relapsing, illness that can fall under the palliative care umbrella due to the potential for significant morbidity and mortality through organ failure, chronic infections, and overdose syndromes. Patients with SUDs have a notably higher risk of all-cause mortality compared to those without (1, 2). Finding exact data on SUD morbidity is challenging given the broad consequences of this disease, however, the economic cost of SUDs in the United States is approximately $3.73 trillion annually, $120 billion of which is from healthcare costs (3). These data highlight the substantial impact of SUDs on both morbidity and mortality, underscoring the need for effective treatment and rehabilitation strategies for this population.

The vulnerable group of interest is comprised of patients with late-stage chronic diseases who have, before or throughout their illness, developed SUDs. Over 46 million Americans over the age of 12 had an SUD in 2021, which equates to 16.5% of the population, with only approximately 6% of those with SUD receiving treatment (4). Since limited research is conducted on this specific population, the number of patients in palliative care with SUD is unknown. Some studies postulate that 6% of palliative care patients also have SUD, while others estimate close to 25% (5).

Substance use disorders often exacerbate existing incurable illnesses, highlighting a critical need for comprehensive palliative care to address not just physical discomfort but also psychosocial distress, existential angst, and sociocultural barriers to care (6). Often, patients with SUD, whether current users or in remission, have inherently poor coping mechanisms that will potentially be exacerbated by the stress of a new diagnosis or symptom, especially one that may be life-threatening or incurable. Combining the intricacies of addiction with incurable illnesses presents a multifaceted scenario that demands innovative solutions and an unwavering commitment to patient-centered care. Currently, there is no consensus or best practice guidelines for management of patients with SUD who need palliative care services.

The National Consensus Project Guidelines for Quality Palliative Care outlined eight key domains, six of the most relevant which are highlighted below (7):

1. *Structure and process of care:* Palliative care principles, integrated across healthcare settings and delivered by all clinicians. They prioritize comprehensive assessment, patient and family engagement, effective communication, care coordination, and continuity of care, supported by interdisciplinary teams.
2. *Physical aspects of care:* Physical care for seriously ill patients involves understanding patient goals and addressing their physical, functional, emotional, and spiritual needs through symptom management and collaboration among healthcare professionals across care settings.
3. *Psychological and psychiatry aspects of care:* A palliative care interdisciplinary team addresses the psychological and psychiatric needs of seriously ill patients through comprehensive mental health screenings facilitated by social workers who provide assessment, treatment, support, and referrals. This ensures patient and family understanding and access to appropriate care and resources.
4. *Social aspects of care:* Palliative care recognizes the significant impact of social determinants of health on patients with serious illnesses. It addresses environmental and social factors to support patient and family functioning and quality of life through identifying strengths and needs facilitated by a professional social worker within the interdisciplinary team.
5. *Spiritual, religious, and existential aspects of care:* Spirituality, integral to compassionate palliative care, encompasses individuals' quest for meaning and connection, expressed through beliefs and practices, with the interdisciplinary team honoring and respecting each patient and family's spiritual preferences and boundaries.
6. *Cultural aspects of care:* The first step in culturally sensitive palliative care involves assessing and respecting values, beliefs, and traditions while continuously expanding awareness of biases and perceptions, culminating in a comprehensive care plan tailored to meet the diverse needs of patients and families, including respectful acknowledgment and support for grieving practices.

There exists a need to advocate for the incorporation of palliative rehabilitation as a holistic strategy to enhance the quality of life for patients with SUD facing incurable diseases. Palliative rehabilitation encompasses a range of interventions, from not only pain and symptom management, but also psychological and social support, tailored to the individual's unique circumstances. Palliative rehabilitation is uniquely positioned to assist in the palliative management of those with SUDs as it integrates principles of palliative care and rehabilitative medicine, which addresses psychosocial and spiritual needs and incorporates harm reduction and addiction treatment strategies (8, 9).

Through these efforts, rehabilitation professionals can aim to alleviate suffering, enhance functionality, and empower individuals to make the most of their remaining time, regardless of the complexities they face due to SUDs and incurable diseases. There is a critical need for a patient-centered and holistic approach to palliative care that can be provided by palliative rehabilitation to address the unique challenges faced by patients with SUD, and improve their overall quality of life and well-being.

Overcoming physical, psychological, and social challenges associated with SUDs is essential for the successful implementation of care, requiring a multidisciplinary approach involving physicians, nurses, social workers, therapists, psychologists, and addiction specialists (10). Building this multifaceted strategy requires a unified front. Palliative rehabilitative efforts can include physical therapy, occupational therapy, speech language pathology, and psychotherapy, alongside comprehensive addiction management strategies, such as medication-assisted treatment and cognitive-behavioral therapy (11, 12). A high priority for these providers should be to align care with the patient's palliative care goals and prioritize quality of life while avoiding increased patient discomfort. This can be best achieved by leveraging and integrating the skills of the entire multidisciplinary team, including physicians, nurses, therapists, psychologists, addiction specialists, and more, to ensure that the approach is truly comprehensive. This underscores the need for a broader recognition of palliative care's potential in enhancing the quality of life for individuals struggling with SUDs and co-existing incurable diseases and including the need for policy changes, specialized training, and system-level reforms to support this vulnerable population better (13). The purpose of this article is to examine the misunderstood and underutilized interaction between palliative care and SUDs and describe unique opportunities provided by physical medicine and rehabilitation providers to achieve a patient's palliative care goals and optimize overall quality of life.

## Challenges with management of SUD in palliative care

### Lack of acknowledgment

All medical conditions deserve identification and recognition by the health care team because each condition, regardless of their nature, can impact an individual's health and quality of life. Proper identification, diagnosis, and recognition are needed so patients receive comprehensive and attentive care to facilitate appropriate treatment and improve patient outcomes. SUD is commonly underrecognized due to stigma and lack of education.

### Stigma behind SUD

A common barrier to healthcare provision is the real or perceived stigma by healthcare providers towards those with SUD. This is likely caused by the misperception that patients with SUD have directly caused their disorder through their own actions differing from other conditions such as depression and other psychiatric disorders (14). There is a robust body of evidence of genetic, physiologic, and social influences on SUD that prompt a disease-oriented or medical approach as opposed to perceiving SUD as a behavioral personality fault (14). Stigma and misperception towards SUD extends beyond healthcare and is likely reinforced by strong negative societal perceptions of SUD, as it is perceived to violate common social and societal norms (15). Psychosocial pressures play a significant role in the development of SUDs as environments where substances are normalized can increase the likelihood of an individual developing a use disorder (16).

A key component of SUD is the biochemical and metabolic changes in the brain caused by consistent use, leading to compulsive behavior (17). Pavlovian learning is a well-defined concept describing how environmental cues associated with drug use become linked with reward anticipation, and this can be used to provide a physiologic basis of cravings and drug-seeking behaviors instead of faulting a patient's “willpower” (17). This process involves neuroplasticity, where synaptic signaling between neurons changes, thereby contributing to learning and memory, which includes the crucial role of dopamine in altering the brain's structure. Drug-induced neuroplastic changes occur in various brain regions implicated in addiction, affecting motivation, craving, and self-regulation (17). Additionally, it is essential to consider the pharmacological concept of drug tolerance. When drugs bind to a receptor in the brain, dopamine levels increase in the body, and a sense of euphoria ensues; however, with continued use, the receptors exhibit reduced responsiveness, termed *tolerance* (18). Tolerance and physiologic dependence can often explain how the use of substances drives compulsive behavior and extends their use. Patients cannot be solely blamed for their substance issues because the development of addiction is multifactorial, involving a complex interplay of genetic, environmental, and neurological factors.

### Limited SUD training

Some healthcare professionals, including palliative care providers, have reported discomfort and lack of role clarity regarding addressing SUD in their patients (19). Additionally, they report low confidence in their ability to effectively address and treat SUD, causing the SUD to be untreated. Providers did report that consultations with addiction specialists would be beneficial for their patients, but this is unfeasible. In 2019, 3,171 physicians were qualified as addiction specialists (e.g., addiction medicine, addiction psychiatry), and it was estimated that 25.7 million people needed palliative care treatment (20). Since there is no concrete data to quantify the number of patients with SUD needing palliative care, this number could range anywhere several millions of people. Simply put, the demand for addiction specialists exceeds the available supply, and it is not feasible to place the care burden solely on them.

Despite efforts to address SUD in palliative care, the field still faces a deficit in education regarding addiction treatment and management. The book *Primer of Palliative Care* is a widely used resource to assist with preparation as a palliative care specialist; within this book there is minimal substantive mention of identification and management of addiction (20). When treating pain with opioids, it states “addictive behavior is a real risk when addiction has been a problem in the past” but should not deter from using opioids in pain treatment and goes on to define addiction, physical dependence, tolerance, and pseudoaddiction (21). A survey of palliative care fellows revealed that the majority lacked confidence in their ability to treat patients with SUD due to limited training (19, 22). Additionally, only 21.1% of fellows reported satisfaction with their skills in managing pain for patients with SUD (19, 23). This insufficient training poses significant risks to the well-being and safety of patients. Many patients in palliative care have terminal conditions related to SUD, emphasizing the importance of addressing their needs (24, 25). Given that the primary goal of palliative care is to enhance quality of life, it is crucial to recognize that patients with a history of SUDs will have negative impacts on their physical and mental health as well as social relationships. These principles align with the core values of palliative care, suggesting that the treatment of SUD falls within its scope. Patients with SUD represent a subset of those in palliative care who require a comprehensive approach by palliative care providers to address both their palliative care and SUD needs effectively. The lack of consensus on how to treat this subset of patients is a major barrier, as well are the lack of resources and expertise. Therefore, changes must be implemented to enhance confidence and capability among all palliative care providers and interdisciplinary team members in managing SUD-related concerns.

## The case for palliative rehabilitation

In addition to the already complex management of non-curable conditions, SUDs also increase the risk of dehumanizing the patient and increasing barriers to empathetic and compassionate care. Beyond physical discomfort, patients face a deluge of psychosocial distress, existential concerns, and sociocultural hurdles (26, 27). The profound effects of SUD, regardless of a patient's current usage, further hamper coping mechanisms, often exacerbating difficulty when coping with a new medical diagnosis.

Palliative rehabilitation provides a unique opportunity to integrate the care approaches of palliative care and rehabilitative medicine. Rehabilitation providers' goals are multifaceted: first to build support networks and connections, and second, to stress the importance of community support groups and connections. A key consideration for those with SUD is connection—connection with their loved ones, community, and support groups. This is critical if the patient is to have any hope of attaining balance within themselves to incorporate any further aspects of care.

There are several studies and approaches related to rehabilitation models for patients with SUDs. These studies have focused on ongoing support and the use of technological aids in managing the population's functional and biopsychosocial aspects (28, 29). Improving the well-being for these patients is a vital aspect of the recovery process. Finally, as this is a unique and novel area of practice, improving the quality and quantity of research on palliative rehabilitation in this population is essential.

## Importance of communication

The relationships between rehabilitation professionals and patients are imperative to treatment outcomes, with communication barriers being a foundational consideration. Providers should utilize lay terms to describe medical conditions to assure patient understanding and control. Additionally, rehabilitation providers should also emphasize using positive expressions and tones to create a supporting and trusting environment for improved patient outcomes (30). This is especially important for this patient population, as the patient may expect that the provider may be judgmental or negative during interactions. Properly utilizing language in communication is another area of importance for providers to help to eliminate stigma and negative connotations for their disease. The National Institute on Drug Abuse (NIDA) created a guide entitled *Words Matter: Preferred Language for Talking About Addiction,* which provides critical strategies to avoid speaking about SUD in a harmful manner (31). The language used in healthcare settings is crucial because it can impact treatment adherence. Therefore, providers must be aware when speaking to this subset of patients. The guide recommends using the diagnosis “substance use disorder” when describing the patient instead of terms like “addict” or “substance abuser” (31). This approach aims to reduce stigma by avoiding placing blame on the patient and acknowledging SUD as a medical condition.

## Patient autonomy and resilience

The patient-centered care approach has been implemented in multiple different healthcare environments to facilitate shared decision-making between the patient and healthcare provider. This form of communication allows for open and honest care preferences that uphold the patient's autonomy. However, this approach has been used less widely in patients with SUD due to prevailing attitudes that people with SUD can be uncooperative, manipulative, or even violent, and they lack the motivation to take control of their conditions. The negative attitudes among health professionals can undermine the patient's sense of empowerment, negatively impacting treatment outcomes (15).

It is imperative to empower patients to regain control of their lives, foster independence, and build a resilient mindset, especially in patients with SUD. Without this empowerment, effectively addressing other SUD variables is less likely, resulting in decreased patient adherence and healthcare utilization (32). Addressing the whole person, not just their substance use, provides the patient with an opportunity to recover autonomy and accountability, leading to more comprehensive care.

## Comprehensive symptom management

Crafting individualized strategies to address pain, physical limitations, and psychological challenges requires thorough assessments. These assessments include an exhaustive physical examination to evaluate for areas of pain and disability, focusing on the neurologic and musculoskeletal elements. Additionally, it is essential to evaluate mental health and coping ability with the patient's current illness and pain. The overarching goal of the assessment is to properly evaluate and predict the patient's current and future functional levels. When combined with physical therapy, this assessment allows for a more comprehensive management of pain, musculoskeletal conditions, and activities of daily living (33).

## Holistic well-being and coordination of interdisciplinary rehabilitation services

In addition to the physical and medical aspects of care, the patient's emotional, spiritual, and psychosocial status must be assessed and supported to achieve a whole-person approach to care. One of the critical domains of palliative medicine is to attempt to comprehensively support the entire patient, including their mind, body, and spirit to have the best chance for alleviation of any and all aspects of their suffering (7). This can include providing direct supportive interventions as well as facilitating the involvement of expert care team members such as pastoral care, integrative medicine, and social work. In addition to emotional and spiritual distress, navigating the healthcare system on an ongoing basis can result in bureaucratic or administrative distress (e.g., multiple appointments, insurance approvals, copays, wait times), and efforts toward mitigating these issues are also essential.

The scope of physical medicine and rehabilitation is vast, with each rehabilitation discipline offering a valuable and unique component within the palliative care team. There is often uncertainty as to the roles of rehabilitation professionals within palliative care as these services are traditionally considered rehabilitative rather than supportive or palliative (34). As many of these patients are facing pain and symptoms that will impact strength, function, and movement ability, physical therapy can improve, maintain, or slow the decline of physical symptoms and assist with safe care transitions (35). Occupational therapy provides a unique and creative perspective in modifying or adapting a patient's activities of daily living and participation in valued life activities (36). Speech-language pathology can assist patients in improving their communication, eating, and drinking skills—all of which are foundational tasks for every human being (37). Additionally, each discipline can provide direct interventions to assist with the patient's cognitive, psychological, emotional, and social status. Although a comprehensive listing of the roles of these rehabilitation professionals within palliative is quite extensive, their proactive involvement in the palliative interdisciplinary team, combined with evidence-based medication-assisted treatments and cognitive-behavioral therapies, can help facilitate patients in palliative care with SUD the best chance to have a good quality of life (34).

## Addiction management

A cornerstone in the management of SUD treatment is medication-assisted treatment (MAT), which incorporates harm reduction and addiction treatment measures tailored to the individual's unique journey. Widely considered to be the standard of care for SUD, numerous research studies demonstrate evidence of the efficacy of MAT for treating addiction and preventing relapse (38, 39). The five goals of MAT include improving patient survival, increasing retention in treatment, decreasing opiate use, increasing patients’ ability to gain and maintain employment, and improving the birth outcome for women who have SUD (40). MAT can assist with symptom management and relapse prevention, commonly through the strategic use of medications methadone and buprenorphine.

Integrating addiction medicine skills into palliative care is essential, given the interdisciplinary nature of palliative care and the recognition that treating patients with SUD is integral to comprehensive care. Palliative care providers have described the importance of treating SUD in patients; however, they report feeling ill-equipped to address this aspect of care due to a lack of training or resources specific to managing SUDs in the palliative care setting (19). SUD experts in palliative care have advocated for the inclusion of primary addiction medicine skills such as opioid prescribing, monitoring, and misuse within specialist palliative care training, as these care needs align with existing core competencies for palliative care patient management (41). Taking this a step further, Jones et al. (41) advocated that the philosophy and skills between palliative care and SUD treatment overlap and perhaps are even synonymous. Due to the substantial scope and impact of SUD within the palliative population, these patients would be best served by the increased availability of addiction specialists, as well as increasing the confidence and competence of palliative care specialists in managing SUD during their advanced specialty training and fellowships.

## Health implications and the downstream effects of SUD

Substance use disorders rarely manifest in isolation and can have downstream implications for patients' health and well-being. Chronic substance use can be a precipitating factor for cardiovascular, hepatic, and renal dysfunction, including end-stage renal disease (ESRD) (24, 25). Consequences of a patient's SUD can have implications beyond palliative care, including complex decisions regarding the impact of substance use on potential treatments, social determinants of health, and considerations regarding organ transplantation. As discussed above, these considerations for palliative care patients with SUD also involve challenges which include societal stigma, legal barriers, potential misuse of prescribed medications, and an ill-equipped healthcare system (42). Thus, comprehensive, interdisciplinary strategies and guidelines must be developed to integrate palliative care in managing patients with SUD (40). The traditional mentality of disregarding addiction in terminal illnesses may exacerbate both the addiction and associated distress, necessitating a shift in clinical culture. Measures must be taken to address the SUD by using addiction management skills (including MAT) to prevent exacerbation of these chronic or life-threatening conditions.

## Advocacy, policy, and training

Advocacy at every level—from community awareness campaigns to policy dialogues—is paramount to bringing about palpable change. Policymakers need to revisit and revise frameworks that inadvertently alienate or stigmatize people with SUD. Furthermore, in order to leverage the unique skills of rehabilitation professionals, healthcare providers require advanced education and specialized training in the key concepts and benefits of palliative rehabilitation.

Currently, many medical schools and post-graduate programs have not integrated SUD education into their curriculum even though the opioid epidemic and SUD remain major public health concerns (7). Healthcare is an ever-changing landscape, and there is a need for specialist palliative care services to adopt primary addiction medicine skills to provide effective and comprehensive care (40).

## Research and future directions

There still lies a substantial gap in the literature regarding consensus guidelines and evidence-based treatment approaches when considering rehabilitation involvement for patients in palliative care with SUD. The few research studies have shown that the intersection of SUD and palliative care is challenging, given the distinct needs of this patient population. This paper suggests important steps based on our clinical practice experience, but more comprehensive clinical research studies are imperative. These studies should evaluate the impact of interventions and describe the nuances of care in order to establish clinical best practices. Such endeavors would not only enrich clinical practice but also inform policy, ensuring sustainable regulations, legislation, and payment.

## Conclusion

Substance use disorder is a complex, multifaceted problem, especially within the context of chronic or life-threatening illness requiring palliative care. Societal biases, rooted in misinformation and stigma, negatively impact care delivery. Legal and regulatory guidelines, often not developed with such complex intersections in mind, may pose barriers to care for these patients. Furthermore, risks and concerns from considerations such as the misuse of prescription medications add another layer of complexity. The current healthcare system is ill-prepared to cater to the needs of these patients due to the lack of guidelines outlining comprehensive care measures.

Palliative rehabilitation provides an opportunity to assist in transforming care for patients with incurable diseases and SUD. Palliative rehabilitation may be able to significantly enhance the quality of life for patients with SUD by providing comprehensive care that addresses both the symptoms of the illness and the addiction itself. We advocate for palliative care providers to identify SUD in their patients, pursue methods to improve their confidence and abilities in providing basic SUD treatment, and proactively involve rehabilitation professionals to provide supportive interventions. These actions, taken in combination, can improve the care team's ability to provide a holistic, patient-centered approach which can have substantial positive outcomes for patients, health systems, and society.

## Data Availability

The original contributions presented in the study are included in the article/Supplementary Material, further inquiries can be directed to the corresponding author.
